# The Association Between the Triglyceride–Glucose Index and the Risk of Diabetic Kidney Disease in Patients with Type 2 Diabetes Mellitus: A Cross-Sectional Study

**DOI:** 10.3390/life16020345

**Published:** 2026-02-17

**Authors:** Munther S. Momani, Raneem Dalaeen, Dia Sarhan, Zaid Sarhan, Suhib Awamleh, Yazan M. Momani, Omar Abu Farsakh

**Affiliations:** Department of Internal Medicine, Faculty of Medicine, The University of Jordan, Queen Rania Street, Amman 11942, Jordansuhib.awamleh@gmail.com (S.A.);

**Keywords:** triglyceride–glucose index, diabetic kidney disease, nephropathy, diabetes mellitus

## Abstract

This study aimed to evaluate the association between the triglyceride–glucose index (TyG) and the risk of diabetic kidney disease (DKD) in patients with type 2 diabetes mellitus. Methods: This study included 1347 patients with type 2 diabetes who attended the endocrinology clinic at Jordan University Hospital between May 2025 and October 2025. Medical records were reviewed to identify patients with documented DKD, and the TyG index was calculated for each patient. Results: Our results showed that patients with both late-stage kidney disease (mean 9.47 ± 0.74) and early-stage kidney disease (mean 9.42 ± 0.67) demonstrated elevated TyG index values compared to those without kidney disease (mean 9.27 ± 0.70). In the fully adjusted model, the association remained robust with an OR of 1.611 (95% CI: 1.330–1.951, *p* < 0.001), indicating that higher TyG index values are independently associated with increased kidney risk even after controlling for major confounding variables. When comparing TyG index quartiles, the second quartile showed no significant difference from the reference group, while the third quartile showed 66% increased odds (OR = 1.66, 95% CI: 1.176–2.345, *p* = 0.004) and the fourth quartile demonstrated 117% increased odds (OR = 2.174, 95% CI: 1.512–3.125, *p* < 0.001). The association between the TyG index and DKD was more significant in patients younger than 60 years, and in women. In conclusion, the TGI was associated with increased risk of DKD; however, its discriminative ability was modest (AUC 0.57). This indicates that the TGI alone is insufficient as a predictive tool and should be interpreted alongside established screening tools. Prospective studies are needed to clarify its causal role in DKD development.

## 1. Introduction

Diabetes mellitus (DM) is a chronic metabolic disorder characterized by persistent hyperglycemia resulting from defects in insulin secretion, insulin action, or both [1]. Globally, the burden of type 2 diabetes mellitus (T2DM) continues to rise, and this trend is particularly evident in Jordan, where the prevalence has shown a steady increase, likely influenced by socioeconomic and lifestyle factors [2]. Older age and lower educational attainment have been significantly associated with a higher prevalence of diabetes in the Jordanian population [3]. Diabetic nephropathy (DN), also referred to as diabetic kidney disease (DKD), develops in approximately 20–40% of patients with diabetes worldwide and represents the leading cause of end-stage renal disease (ESRD) [4]. In Jordan, evidence indicates that nearly half of all patients with T2DM develop DKD [5]. Another study conducted at the National Diabetes Center in Jordan reported that approximately 33% of patients with diabetes had nephropathy [6]. The economic burden of diabetic nephropathy and ESRD is massive. In the United States, Medicare spending on chronic kidney disease (CKD) and ESRD exceeded $120 billion in 2017, of which $49 billion was spent on ESRD management alone [7]. Similarly, in Jordan, the annual cost of hemodialysis at Ministry of Health hospitals reached USD 17.7 million, averaging USD 9976 per patient, representing approximately 2.7% of the national health budget [8]. Pathophysiologically, diabetic nephropathy results from chronic hyperglycemia-induced vascular injury affecting glomerular capillaries and arterioles. It is characterized by progressive proteinuria, declining renal function, and eventual progression to ESRD [9]. Diagnosis typically involves the urinary albumin test, albumin-to-creatinine ratio, and estimation of glomerular filtration rate (GFR), supplemented by imaging or biopsy when needed [10,11]. Early detection and intervention are therefore crucial for reducing mortality and disease burden [12]. The triglyceride–glucose index (TyG), a composite biomarker derived from fasting triglyceride and glucose levels, has emerged as a reliable surrogate for insulin resistance due to its high sensitivity and specificity [13]. Recent studies have identified a positive association between higher TyG index values and renal function decline, with patients in the highest TyG index tertile demonstrating a significantly increased risk of developing DKD [14,15]. Furthermore, the TyG index may serve as an early predictor of kidney injury in patients with T2DM [16]. Despite these findings, evidence on the relationship between the TyG index and diabetic nephropathy remains limited, particularly on variations across age and gender groups. Moreover, there is a notable lack of research on this association within Middle Eastern populations, whose unique genetic, metabolic, and lifestyle characteristics may influence the risk and progression of DKD. Given this context, the present study aims to investigate the association between the triglyceride–glucose index and the risk of diabetic nephropathy among patients with type 2 diabetes mellitus in Jordan, thereby addressing an important gap in regional data and contributing to improved understanding of metabolic predictors of renal complications.

## 2. Methods

### 2.1. Study Design and Population

A cross-sectional study was conducted at a single tertiary care center in the outpatient endocrinology and nephrology clinics at Jordan University Hospital (JUH) between May 2025 and October 2025. A total of 1414 adult patients diagnosed with type 2 diabetes mellitus (T2DM), aged 30–85 years, were assessed for eligibility. Participants were enrolled regardless of the presence or absence of kidney-related complications. In total, 67 patients were excluded because they had missing data required to ascertain major adverse cardiovascular events (MACE), insufficient information to calculate the TyG index, or if they declined to provide informed consent. After these exclusions, 1347 participants met all eligibility requirements and were included in the final analysis. The study protocol was reviewed and approved by the Institutional Review Board (IRB) of Jordan University Hospital (10/2025/10387 dated 5 May 2025), and all procedures complied with relevant ethical standards.

### 2.2. Assessment of Triglyceride–Glucose Index

The triglyceride–glucose index (TyG) was calculated using the following formula: TyG = Ln [fasting triglycerides (mg/dL) × fasting glucose (mg/dL)/2] [17]. Both triglyceride and fasting glucose concentrations were determined via an enzymatic assay using an automated biochemistry analyzer (Atellica CI Analyzer, Siemens Healthineers AG, Forchheim, Germany) in the hospital’s central laboratory.

### 2.3. Assessment of Nephropathy

Renal function and nephropathy status were evaluated using both glomerular filtration and albumin excretion markers. Albuminuria was assessed using the urine albumin-to-creatinine ratio (UACR), and patients were categorized as normoalbuminuria (UACR < 30 mg/g), microalbuminuria (UACR 30–299 mg/g), or macroalbuminuria (UACR ≥ 300 mg/g). Estimated glomerular filtration rate (eGFR) was calculated using the Modification of Diet in Renal Disease (MDRD) equation. The presence of chronic kidney disease (CKD) was determined based on prior documentation in the patients’ medical records by treating nephrologists at the nephrology clinic. Laboratory parameters obtained at the time of study assessment were used for classification and staging purposes. For analytical purposes, clinically significant renal involvement was defined as eGFR < 90 mL/min/1.73 m^2^ and/or UACR ≥ 300 mg/g. The use of UACR ≥ 300 mg/g as part of the operational definition aimed to identify patients with more advanced renal impairment. However, we acknowledge that this threshold may not fully capture early-stage diabetic kidney disease, particularly patients with isolated microalbuminuria (UACR 30–299 mg/g). Given the cross-sectional design, renal staging was based on measurements obtained at a single time point. Although CKD diagnosis reflected prior clinical documentation, persistence of abnormalities for ≥3 months could not be independently verified for all participants within the study framework. All laboratory investigations were performed at the JUH Central Laboratory in accordance with standard operating procedures and quality control protocols.

### 2.4. Assessment of Diabetes

DM was defined as either being on pharmacological treatment for diabetes mellitus or having a diagnosis with hemoglobin A1c ≥ 6.5%, fasting plasma glucose (FPG) ≥ 126 mg/dL, or a 2 h blood glucose ≥ 200 mg/dL after a 75 mg glucose load consistent with the American Diabetes Association guidelines [18].

### 2.5. Section of Covariates

Data on various demographic and health-related factors were collected through the medical record system at Jordan University Hospital by trained clinical staff. The collected covariates included age, gender, duration of diabetes, presence of diabetic complications (such as neuropathy, nephropathy, and retinopathy), smoking status, body weight, body mass index (BMI), and systolic and diastolic blood pressure. Laboratory measurements included hemoglobin A1c, serum creatinine, low-density lipoprotein cholesterol (LDL-C), high-density lipoprotein cholesterol (HDL-C), triglycerides, vitamin D, parathyroid hormone, serum calcium, serum phosphorus, albumin, alkaline phosphatase, and magnesium. Renal function was assessed using the estimated glomerular filtration rate (eGFR) calculated through the Modification of Diet in Renal Disease (MDRD) equation. Additionally, medication history was documented, including the use of anti-diabetic agents (metformin, insulin, and other oral hypoglycemics), aspirin, angiotensin receptor blockers (ARBs), angiotensin-converting enzyme inhibitors (ACEIs), statins, beta blockers, diuretics, calcium channel blockers (CCBs), and proton pump inhibitors (PPIs).

### 2.6. Data Analysis

All statistical analyses were conducted using SPSS (version 27). Distributional properties of continuous variables were assessed using visual inspection of histograms and Q–Q plots. Continuous variables are presented as means with standard deviations for approximately normally distributed variables and as medians with interquartile ranges for non-normally distributed variables, while categorical variables are presented as counts and percentages. Two-sided *p*-values <0.05 were considered statistically significant. Baseline demographic, clinical, medication, and laboratory characteristics were summarized for the entire cohort and compared between patients with kidney disease (early and late stages combined) and those without kidney disease. Continuous variables (e.g., age, body mass index, diabetes duration, and laboratory measures) were compared using independent samples t-tests for normally distributed variables and Mann–Whitney U for non-normally distributed variables, while categorical variables (e.g., sex, smoking status, hypertension, and medication use) were compared using chi-square tests. When kidney disease was modeled as a three-level categorical variable (no kidney disease, early-stage disease, and late-stage disease), differences in continuous variables across these groups were assessed using one-way analysis of variance (ANOVA). When the overall ANOVA was statistically significant, post hoc pairwise comparisons were performed to identify differences between specific disease stages. Additional descriptive comparisons were conducted after stratifying participants by sex and by age group (<60 years vs. ≥60 years), using independent samples t-tests for continuous variables and chi-square tests for categorical variables. The association between the TyG index and kidney disease was examined using multiple complementary approaches. First, binary logistic regression was used with kidney disease (early or late stage vs. no disease) as the dependent variable and the TyG index entered as a continuous predictor. Three models were constructed: an unadjusted model, a model adjusted for demographic variables (sex, age, body mass index, and smoking status), and a fully adjusted model additionally including hypertension and HbA1c. To assess potential non-linear or threshold effects, the TyG index was also categorized into quartiles and entered as a categorical predictor in logistic regression models, with the lowest quartile serving as the reference group. The same stepwise adjustment strategy was applied. To evaluate whether the TyG index was associated not only with disease presence but also with disease severity, ordinal logistic regression was performed with kidney disease stage (no disease, early stage, or late stage) treated as an ordered outcome. The proportional odds framework was used, and the same sequence of unadjusted, demographic-adjusted, and fully adjusted models was fitted. In addition, restricted cubic spline (RCS) functions were applied to further characterize the shape of the association between the TyG index (modeled as a continuous variable) and kidney disease outcomes. Multivariable logistic regression models incorporating RCS terms were fitted to examine (1) kidney disease of any stage versus no disease, (2) early-stage kidney disease versus no disease, and (3) late-stage kidney disease versus no disease. Four knots were placed at default quantiles, and overall associations as well as departures from linearity were assessed using Wald χ^2^ tests. Odds ratios and 95% confidence intervals were estimated relative to the median TyG index value. RCS analyses were conducted using R software (version 4.5.0) with the rms package. Receiver operating characteristic (ROC) curve analysis was used to assess the discriminatory performance of the TyG index for identifying kidney disease. The area under the curve (AUC) was calculated, and the optimal cut-off point was determined based on the balance between sensitivity and specificity. Finally, subgroup analyses were conducted to explore effect modification by sex and age. Separate fully adjusted logistic regression models were fitted within strata defined by sex (male vs. female) and age (<60 vs. ≥60 years), using kidney disease as the outcome and the TyG index as the predictor, adjusted for the same covariates as in the primary fully adjusted model.

## 3. Results

### 3.1. Characteristics of the Included Patients

A total of 1347 patients with diabetes were included in the analysis, of whom 593 (44.0%) had late and early-stage kidney disease. Table 1 presents the baseline characteristics of the study population stratified by kidney disease status. Patients with late and early-stage kidney disease were significantly older than those without kidney disease, with mean ages of 64.04 and 56.32 years respectively (*p* < 0.001). Similarly, diabetes duration was significantly longer in the kidney disease group, averaging 12.64 years compared to 9.00 years in those without kidney disease (*p* < 0.001). The prevalence of hypertension was markedly higher among patients with kidney disease, affecting 80.8% compared to 60.7% in the non-disease group (*p* < 0.001). Current smoking was more prevalent in the kidney disease group (12.6% versus 20.3%), though ex-smokers were more common among those with kidney disease (15.3% versus 11.2%, *p* < 0.001). Medication use patterns differed substantially between groups, with patients with kidney disease showing higher utilization of aspirin, beta blockers, diuretics, metformin, calcium channel blockers, and proton-pump inhibitors, while angiotensin receptor blocker use was lower in this group (all *p* < 0.001). Laboratory parameters revealed significantly elevated creatinine (1.27 versus 0.63 mg/dL), reduced CKD score (63 versus 102.29), and markedly increased PTH levels (108.68 versus 63.6 pg/mL) in patients with kidney disease (all *p* < 0.001). Gender distribution, BMI, HbA1c, and use of ACE inhibitors and statins did not differ significantly between the two groups.

Table 2 shows the baseline demographic and clinical characteristics stratified by sex. Females had a significantly higher mean body mass index compared with males, whereas males exhibited higher serum creatinine levels. No significant differences were observed between males and females with respect to diabetes duration or HbA1c levels. Smoking status differed significantly by sex, with a higher proportion of current and former smokers among males. In addition, hypertension was more prevalent among females than males.

Table 3 shows the baseline demographic and clinical characteristics stratified by age group (<60 years vs. ≥60 years). Participants aged ≥60 years demonstrated significantly higher serum creatinine levels and longer diabetes duration compared with those aged <60 years. Conversely, HbA1c levels and body mass index were higher among participants aged <60 years. Significant differences in smoking status and hypertension prevalence were also observed between the two age groups, with higher rates of hypertension among older participants.

### 3.2. TyG Index and DKD

Table 4 shows the association between the TyG index and DKD stages. The overall ANOVA revealed significant differences across groups (*p* < 0.001). Patients with both late-stage kidney disease (mean 9.47 ± 0.74) and early-stage kidney disease (mean 9.42 ± 0.67) demonstrated elevated TyG index values compared to those without kidney disease (mean 9.27 ± 0.70). Post hoc comparisons confirmed that both late-stage (*p* < 0.001) and early-stage (*p* = 0.004) groups had significantly higher TyG index values than the no kidney disease group. The difference between late and early-stage disease was not significant (*p* = 0.684), suggesting that TyG index elevation occurs early in kidney disease and remains stable across disease progression.

Table 5 presents the logistic regression analysis examining the TyG index as a continuous predictor of kidney disease presence. In the unadjusted model, each unit increase in the TyG index was associated with 39% increased odds of having kidney disease (OR = 1.391, 95% CI: 1.191–1.625, *p* < 0.001). After adjusting for demographic factors including sex, age, body mass index, and smoking status in Model 2, the association strengthened to an odds ratio of 1.597 (95% CI: 1.344–1.899, *p* < 0.001). In the fully adjusted model accounting for hypertension and HbA1c in addition to demographic variables, the association remained robust with an OR of 1.611 (95% CI: 1.330–1.951, *p* < 0.001), indicating that higher TyG index values are independently associated with increased kidney disease risk even after controlling for major confounding variables.

Table 6 shows the association between TyG index quartiles and kidney disease, with the first (lowest) quartile serving as the reference group. In the unadjusted model, patients in the third quartile showed a 52.5% increased odds of kidney disease compared to the first quartile (OR = 1.525, 95% CI: 1.121–2.073, *p* = 0.007), while those in the fourth (highest) quartile demonstrated a 73.7% increased odds (OR = 1.737, 95% CI: 1.278–2.362, *p* < 0.001). The second quartile showed no significant difference from the reference group. After demographic adjustment in Model 2, the associations strengthened substantially, with the fourth quartile showing more than double the odds of kidney disease (OR = 2.179, 95% CI: 1.553–3.056, *p* < 0.001). In the fully adjusted model, these associations persisted, with the third quartile showing 66% increased odds (OR = 1.66, 95% CI: 1.176–2.345, *p* = 0.004) and the fourth quartile demonstrating 117% increased odds (OR = 2.174, 95% CI: 1.512–3.125, *p* < 0.001).

Table 7 presents ordinal regression analysis modeling kidney disease severity as an ordered outcome (late stage, early stage, no disease). The TyG index showed a significant inverse association with favorable kidney health status across all models. In the unadjusted model, each unit increase in TyG index was associated with 29% reduced odds of being in a better kidney health category (OR = 0.713, 95% CI: 0.615–0.827, *p* < 0.001). After demographic adjustment, the association strengthened with an OR of 0.614 (95% CI: 0.521–0.723, *p* < 0.001). The fully adjusted model yielded an OR of 0.609 (95% CI: 0.509–0.729, *p* < 0.001), indicating that higher TyG index values are associated with more severe kidney disease stages, even after accounting for demographic factors, hypertension, and glycemic control. This ordinal analysis complements the binary logistic regression by demonstrating that TyG index is associated not only with kidney disease presence but also with disease severity.

Table 8 shows the association between TyG Index and CKD outcomes using restricted cubic spline models. In multivariable logistic regression analyses using restricted cubic splines, the triglyceride–glucose (TyG) index was significantly associated with chronic kidney disease (CKD) across disease stages. For CKD of any stage, the TyG index demonstrated a strong overall association (overall *p* < 0.0001) with no evidence of nonlinearity (*p* for nonlinearity = 0.44), indicating a predominantly linear relationship; relatively to the median TyG index value, individuals at approximately the 75th percentile (TyG ≈ 9.75) had higher adjusted odds of CKD (OR 1.39, 95% CI 1.16–1.67). Stage-specific analyses showed that higher TyG index was also associated with early-stage CKD (overall *p* = 0.0026; *p* for nonlinearity = 0.62), with a 1.27-fold increase in adjusted odds at the 75th percentile (TyG ≈ 9.71; OR 1.27, 95% CI 1.03–1.57). The association was more pronounced for late-stage CKD, where the TyG index exhibited a strong overall association (overall *p* < 0.0001; *p* for nonlinearity = 0.30), and individuals at the 75th percentile (TyG ≈ 9.72) had substantially higher adjusted odds of late-stage disease (OR 1.56, 95% CI 1.20–2.04). Collectively, these findings indicate that elevated TyG index is associated with CKD across the disease spectrum, with progressively stronger associations observed in more advanced stages.

**Figure 1:**
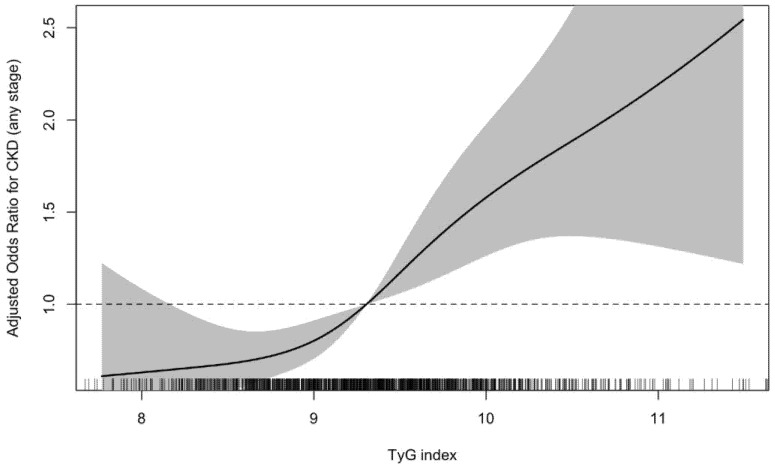
Adjusted association between the TyG index and chronic kidney disease.

**Figure 2:**
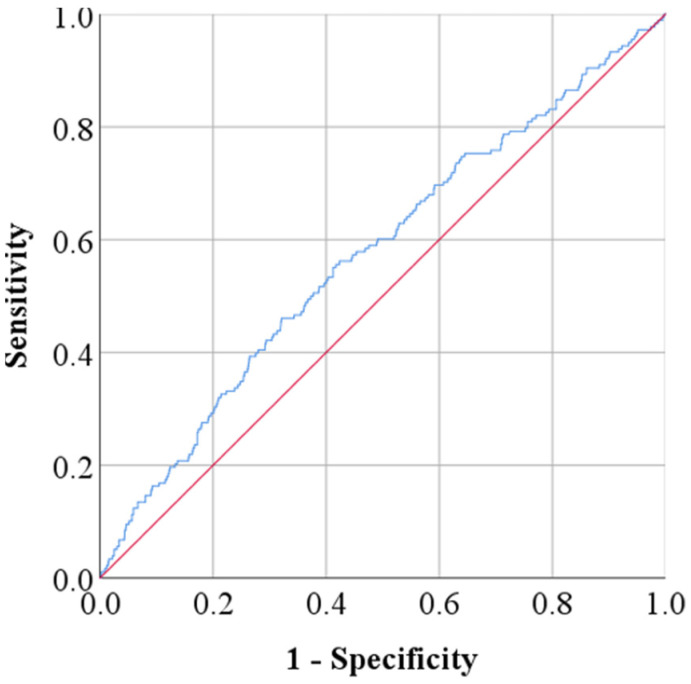
ROC curve for the TyG index with an area under the curve (AUC) of 0.57.

Figure 1 illustrates the restricted cubic spline depicting the adjusted association between the triglyceride–glucose (TG-Glu) index and odds of chronic kidney disease (any stage). The solid line represents adjusted odds ratios relative to the median TG-Glu value, and the shaded area indicates 95% confidence intervals. As shown in Figure 2, odds of CKD increased progressively with higher TG-Glu values; confidence intervals crossed unity around the median and remained entirely above 1 at higher TG-Glu levels, indicating significantly increased odds of CKD at elevated TG-Glu values.

ROC analysis demonstrated that TG-Glu index of 9.36 and higher had an AUC of 0.57 (Figure 1) (*p* = 0.001) with 57% sensitivity and 55% specificity (Table 9).

### 3.3. Age and Sex Subgroup Analysis

Table 10 shows the association between the TyG index and kidney disease (combined early and late stages) stratified by sex and age group. When stratified by sex, the relationship between the TyG index and kidney disease was notably stronger among females compared to males. In females, each unit increase in the TyG index was associated with an 83% increased odds of kidney disease (OR = 1.831, 95% CI: 1.417–2.365, *p* < 0.001), while in males the association was weaker and approached but did not reach statistical significance (OR = 1.328, 95% CI: 0.986–1.788, *p* = 0.062). This suggests that the metabolic dysregulation captured by the TyG index may have differential implications for kidney disease risk between sexes. Age stratification revealed that the TyG index was significantly associated with kidney disease in both younger and older patients, though the association appeared somewhat stronger in those under 60 years of age (OR = 1.745, 95% CI: 1.341–2.271, *p* < 0.001) compared to those aged 60 and above (OR = 1.526, 95% CI: 1.153–2.020, *p* = 0.003). These findings indicate that while the TyG index is a relevant predictor across age groups, the strength of its association with kidney disease may be modified by both sex and age, with particularly strong effects observed in females and younger individuals.

## 4. Discussion

Our study shows a significant association between the TyG index and the prevalence and severity of diabetic kidney disease (DKD) in a cross-sectional cohort of Jordanian adults with type 2 diabetes. Nearly 44% of participants exhibited early or late DKD, and the mean TyG index values were substantially higher in those with DKD. In multivariable models, each one unit increase in TyG index value was associated with roughly 61% higher odds of having DKD after adjusting for sex, age, body mass index (BMI), smoking status, hypertension, and HbA1c. Stratifying the TyG index into quartiles showed that individuals in the highest quartile had approximately twice the risk of DKD compared with those in the lowest quartile, and ordinal regression indicated that higher TyG index values corresponded to more severe stages of DKD. A receiver operating characteristic-derived cut-off of about 9.36 provided the best balance of sensitivity and specificity; although the area under the curve was modest (0.57), crossing this cut-off corresponded to an approximately 1.4-fold increase in the odds of any-stage DKD, and roughly 1.6-fold for late-stage DKD. These patterns were most pronounced in women and participants younger than 60 years, whereas the association was weaker in men and older adults. Insulin resistance (IR) is the principal pathophysiological link between the TyG index and renal injury. Elevated fasting triglycerides and glucose reflect impaired glucose up-take and dysregulated lipid metabolism; this state induces glomerular hyperfiltration by dilating afferent arterioles and increasing renal plasma flow [19]. This hyperfiltration raises intraglomerular pressure and accelerates basement membrane thickening and mesangial expansion, culminating in albuminuria and progressive nephron loss. Excess adiposity and insulin resistance also reduce membrane thickening and mesangial expansion, culminating in albuminuria and progressive nephron loss. Excess adiposity and insulin resistance also reduce nitric oxide bioavailability, heightening sodium retention and activating the RAAS [20]. Elevated angiotensin II and aldosterone promote glomerular hypertrophy, fibrosis and inflammation, while oxidative stress damages podocytes and tubular cells. These mechanistic pathways provide biological plausibility for the observed association between higher TyG index and increased nephropathy risk. The threshold effect may reflect the point at which compensatory mechanisms are overwhelmed and pathological processes accelerate [21]. Previous studies have also reported an association between higher TyG index values and acute kidney injury, including contrast-induced nephropathy following percutaneous coronary intervention [22]. Although acute renal injury was not evaluated in our cohort, these findings further support the broader concept that insulin resistance-related metabolic dysfunction may increase renal susceptibility.

The observed stronger association between the TyG index and DKD in women corroborates the existing literature suggesting that women may be more vulnerable to insulin resistance-mediated renal injury. An International Journal of General Medicine study found that elevated TyG index quartiles were independently associated with impaired renal function only in women aged ≥ 50 years [23]. Proposed mechanisms include postmenopausal decline in estrogen, which increases visceral adiposity and insulin resistance, heightened inflammatory responses, and menopausal decline in estrogen, which increases visceral adiposity and insulin resistance, heightened inflammatory responses, and activation of the renin–angiotensin–aldosterone system (RAAS) [24,25]. With respect to age, our data showed that the association between the TyG index and DKD was strongest in participants aged < 60 years and attenuated in those aged ≥ 60 years. Our finding is consistent with the results of a nationwide longitudinal study of >1 million Korean adults with type 2 diabetes, as the authors reported that the TyG index was strongly associated with future development of end-stage renal disease (ESRD) and that this association was markedly stronger in participants younger than 50 years [26]. Other studies, however, found consistent associations across age strata [27]. A possible explanation is that younger individuals may be in earlier disease stages where metabolic perturbations exert a larger relative effect, whereas older adults may already have advanced nephrosclerosis or competing risk factors that obscure the impact of the TyG index; also, older individuals are more likely to be taking multiple medications and have a greater cardiac and renal burden beyond IR or obesity, which may weaken the prediction of cardiac and renal disease risk outcomes by the TyG index and its obesity metrics. These findings highlight the importance of age- and sex-specific risk assessment when using the TyG index as a biomarker. Our findings align with growing evidence that the TyG index predicts renal dysfunction in diabetic and general populations. A cross-sectional Chinese study reported that each one-unit increase in the TyG index increased the risk of DKD by 1.94-fold once the index exceeded a threshold of 9.35 [28]. Similarly, a Moroccan cohort found that a TyG index cut-off of 9.58 doubled the odds of nephropathy and achieved 71% specificity [29]. The cut-off of 9.36 identified in our cohort is nearly identical to these thresholds, while differences in study design, ethnicity, and DKD definitions limit direct comparability across populations. Consistent with this pattern, crossing the TyG index cut-off of 9.36 in our cohort corresponded to an approximately 1.4-fold increase in the odds of any-stage DKD compared to the median TyG index value, rising to about 1.6-fold for late-stage DKD, while participants in the highest quartile (≈9.45 and above) exhibited roughly a two-fold increased risk compared to those in the lowest quartile, based on our multivariable analyses; however, given the cross-sectional design, these findings should be interpreted as associative rather than indicative of a universal risk threshold. Evidence for DKD stage-specific TyG index associations remains limited to our knowledge. Most other studies analyze albuminuria categories or composite CKD definitions and seldom consider mildly reduced eGFR or early-stage disease. By defining kidney involvement as eGFR < 90 mL/min/1.73 m^2^ and/or UACR ≥ 300 mg/g, our study captured early stages and showed that risk rises progressively across the spectrum, providing preliminary evidence that the TyG index can detect early renal involvement and potentially enable earlier interventions. This is particularly important in the Middle East region as it has some of the world’s highest rates of type 2 diabetes and metabolic syndrome and Jordan’s prevalence is expected to surpass 20% by 2050 [30]. High intake of refined carbohydrates, obesity, and genetic factors may contribute to elevated baseline TyG index values in Middle Eastern populations [31]. Regional evidence for a DKD stage-specific association with the TyG index is otherwise scarce, with no prior Middle Eastern cohorts analyzing TyG index associations with early and late-stage DKD; concurrently, our study offers the first large-scale assessment of the TyG index and nephropathy in this region, integrates both eGFR and UACR criteria, and demonstrates a linear relationship across disease stages. Given the heavy burden of diabetic nephropathy and limited nephrology resources in many Middle Eastern health systems, the TyG index could serve as a cost effective screening tool, though its predictive value should be validated in multicenter studies across diverse ethnic and socioeconomic groups. The strengths of this study include its relatively large sample size, comprehensive clinical and laboratory data, and rigorous adjustment for multiple confounders. The inclusion of sex- and age-stratified analyses enhances understanding of potential effect modifiers. However, the cross-sectional design precludes causal inference; an elevated TyG index may both contribute to and result from renal dysfunction. Residual confounding from unmeasured factors such as diet, physical activity, medication adherence and specific classes of glucose-lowering therapies, such as SGLT2 inhibitors, cannot be ruled out, and the modest area under the curve highlights limited discriminative power. Finally, the single-center nature of the cohort limits generalizability and the lack of longitudinal data prevents evaluation of whether changes in the TyG index over time predict renal outcomes. Clinically, our findings support incorporating the TyG index into risk-stratification frameworks for diabetic nephropathy. Because the index is inexpensive and derived from routine fasting triglyceride and glucose measurements, clinicians could calculate it during regular patient follow-up visits. Patients with high TyG index values, particularly women, and those under 60 years of age, might benefit from closer monitoring of albuminuria and eGFR, more aggressive blood pressure and glycemic control and early initiation of renin–angiotensin–aldosterone system inhibitors or sodium glucose co-transporter-2 inhibitors. Nevertheless, given its modest predictive accuracy, the TyG index should complement rather than replace established markers such as HbA1c, eGFR and the urinary albumin-to-creatinine ratio. Future research should focus on prospective cohort studies to confirm temporal relationships, evaluate whether interventions that lower the TyG index translate into improved renal outcomes and explore combinations of the TyG index with anthropometric indices or other biomarkers. Mechanistic studies examining how insulin resistance interacts with RAAS activation, oxidative stress and inflammation within the kidney could reveal therapeutic targets for slowing diabetic nephropathy progression.

## 5. Limitation

This study has several limitations that should be acknowledged. First, renal involvement was operationally defined using eGFR < 90 mL/min/1.73 m^2^ and/or UACR ≥ 300 mg/g to identify clinically significant renal impairment. Although albuminuria was categorized into normoalbuminuria, microalbuminuria, and macroalbuminuria, the use of UACR ≥ 300 mg/g as part of the analytical definition may have underrepresented patients with early-stage diabetic kidney disease, particularly those with isolated microalbuminuria (UACR 30–299 mg/g). Consequently, the findings may reflect associations more strongly in patients with more advanced renal involvement. Second, due to the cross-sectional design, renal parameters were assessed at a single time point. Although chronic kidney disease (CKD) status was determined based on prior clinical documentation in patients’ medical records by treating nephrologists, persistence of kidney abnormalities for ≥3 months could not be independently verified within the study framework. Therefore, the study reflects documented CKD and renal impairment at assessment rather than prospective confirmation of chronicity. Finally, as a single-center study conducted at a tertiary care institution, the findings may not be fully generalizable to the broader diabetic population, particularly those managed in non-tertiary settings.

## 6. Conclusions

Our study demonstrated that higher TyG index values are independently associated with the prevalence and severity of diabetic kidney disease. The risk appears to increase markedly once the TyG index exceeds ≈9.36 with stronger associations observed among women and individuals younger than 60 years in subgroup analyses. These findings suggest that the TyG index may serve as a pragmatic, cost effective, though not definitive, tool for identifying patients at elevated risk of DKD. Ongoing research should validate these results in diverse populations and determine whether TyG adds incremental predictive value beyond established renal biomarkers and confirm temporal relationships in prospective cohorts.

## Figures and Tables

**Table 1:** Characteristics of the included patients according to their kidney disease status.

		Kidney Disease		
		No Kidney Disease Count (%) Mean ± SD Median (Q1–Q3)	Late and Early-Stage Disease Count (%) Mean ± SD Median (Q1–Q3)	*p*-Value
Gender	Female	446 (59.2)	344 (58)	0.677
	Male	308 (40.8)	249 (42)	
Age		56.32 + 10.85	64.04 + 9.84	<0.001
BMI		32.760 + 6.65	33.23 + 6.37	0.191
Smoking status	Non-smoker	515 (68.5)	426 (72)	
	Ex-smoker	84 (11.2)	91 (15.3)	<0.001
	Smoker	153 (20.3)	75 (12.6)	
Hypertension	No	296 (39.3)	114 (19.2)	<0.001
	Yes	458 (60.7)	479 (80.8)	
DM duration		9.00 + 6.96	12.64 + 9.04	<0.001
Insulin	No	405 (53.7)	277 (46.7)	0.011
	Yes	349 (46.2)	316 (53.2)	
Mean GFR		125.25 + 29.7	64.39 + 25.95	<0.001
HbA1c		7.71 + 1.58	7.7 + 1.59	0.896
Aspirin	No	251 (33.3)	137 (23.1)	<0.001
	Yes	503 (66.7)	456 (76.9)	
Angiotensin receptor blocker	No	536 (71.1)	337 (56.8)	<0.001
	Yes	218 (28.9)	256 (43.2)	
Angiotensin-converting enzyme inhibitor	No	609 (80.8)	484 (81.6)	0.692
	Yes	145 (19.2)	109 (18.4)	
Statin	No	132 (17.5)	90 (15.2)	0.253
	Yes	622 (82.5)	503 (84.8)	
Beta blockers	No	495 (65.6)	259 (43.7)	<0.001
	Yes	259 (34.4)	334 (56.3)	
Diuretics	No	574 (76.1)	282 (47.6)	<0.001
	Yes	180 (23.9)	311 (52.4)	
Metformin	No	104 (13.8)	195 (32.9)	<0.001
	Yes	650 (86.2)	398 (67.1)	
Calcium channel blockers	No	620 (82.2)	352 (59.4)	<0.001
	Yes	134 (17.8)	241 (40.6)	
Proton-pump inhibitors	No	362 (48)	222 (37.4)	<0.001
	Yes	392 (52)	371 (62.6)	
Oral anti-diabetic agents	No	405 (53.7)	336 (56.7)	0.28
	Yes	349 (46.3)	257 (43.3)	
Creatinine		0.63 + 0.14	1.27 + 0.83	<0.001
CKD score		102.29 + 12.43	63 + 23.05	<0.001
LDL		94.00 (52–136)	87.00 (46–128)	<0.001
HDL		46.15 + 19.96	43.8 + 21.47	0.038
Vitamin d		22.96 + 16.54	26.42 + 28.7	0.002
PTH		63.6 + 29.69	108.68 + 144.64	<0.001
Calcium		9.52 + 0.5	9.43 + 8.56	0.005
Phosphorous		3.50 (2.7–4.3)	3.50 (2.7–4.3)	0.585
Albumin		4.36 + 3.35	4.18 + 2.49	<0.001
Alkaline phosphatase		81.78 + 26.25	92.77 + 77.23	<0.001

**Table 2:** Baseline demographic and clinical characteristics stratified by sex.

Variable	Category	Male	Female	*p*-Value
BMI	Mean ± SD	30.86 ± 5.34	34.449 ± 6.87	<0.001
Creatinine	Mean ± SD	1.04 ± 0.66	0.813 ± 0.62	<0.001
DM duration	Mean ± SD	10.94 ± 8.68	10.36 ± 7.75	0.202
HbA1c	Mean ± SD	7.74 ± 1.59	7.69 ± 1.59	0.63
Smoking status	Non-smoker	260	685	<0.001
	Ex-smoker	141	34	
	Smoker	157	70	
Hypertension	Yes	367	570	0.015
	No	190	220	

**Table 3:** Baseline demographic and clinical characteristics stratified by age.

Variable	Category	Age < 60	Age ≥ 60	*p*-Value
BMI	Mean ± SD	33.40 ± 6.94	32.49 ± 6.00	0.01
Creatinine	Median (Q1–Q3)	0.68 (0.35–1.01)	0.85 (0.36–1.34)	<0.001
DM duration	Mean ± SD	8.60 ± 6.75	12.82 ± 8.94	<0.001
HbA1c	Mean ± SD	7.90 ± 1.69	7.50 ± 1.43	<0.001
Smoking status	Non-smoker	493	452	<0.001
	Ex-smoker	59	116	
	Smoker	152	75	
Hypertension	Yes	429	508	<0.001
	No	277	133	

**Table 4:** The association between TyG index and kidney disease stages.

				ANOVA	Late vs. Early	Late vs. No Kidney Disease	Early vs. No Kidney Disease
Stages of Kidney Disease	N	Mean TyG Index	SD	*p*-Value	*p*-Value	*p*-Value	*p*-Value
Late	24	9.47	0.7				
	5		4	<0.001	0.684	<0.001	0.004
Early	33	9.42	0.6				
	5		7				
No Kidney Disease	79	9.27	0.7				
	1		0				

SD = standard deviation; TyG index: Triglyceride to glucose index.

**Table 5:** Regression analysis of the association between TyG index and kidney disease.

Model 1: Unadjusted			Model 2: Demographic Adjusted		Model 3: Fully Adjusted	
Variable	OR (95% CL)	*p*	OR (95% CL)	*p*	OR (95% CL)	*p*
TyG index	1.391 (1.191–1.625)	<0.001	1.597 (1.344–1.899)	<0.001	1.611 (1.330–1.951)	<0.001

Model 2 = adjusted for sex, age, body mass index, and smoking status. Model 3 = adjusted for Model 2 + hypertension, and HbA1c. TyG index: Triglyceride to glucose index.

**Table 6:** Regression analysis of the association between TyG index per quartile and kidney disease.

Model 1: Unadjusted			Model 2: Demographic Adjusted		Model 3: Fully Adjusted	
Variable	OR (95% CL)	*p*	OR (95% CL)	*p*	OR (95% CL)	*p*
First Quartile	Ref		Ref		Ref	
Second Quartile	1.1 (0.807–1.501)	0.547	1.141 (0.816–1.597)	0.441	1.146 (0.817–1.607)	0.43
Third Quartile	1.525 (1.121–2.073)	0.007	1.678 (1.203–2.341)	0.002	1.66 (1.176–2.345)	0.004
Fourth Quartile	1.737 (1.278–2.362)	<0.001	2.179 (1.553–3.056)	<0.001	2.174 (1.512–3.125)	<0.001

Model 2 = adjusted for sex, age, body mass index, and smoking status. Model 3 = adjusted for Model 2 + hypertension, and HbA1c. TyG index: Triglyceride to glucose index.

**Table 7:** Ordinal regression analysis of TyG index and kidney disease severity.

	OR	*p*-Value	95% Confidence Interval	
			Lower Bound	Upper Bound
Model 1: Unadjusted	0.713195	<0.001	0.615082	0.826959
Model 2: Demographic adjusted	0.613853	<0.001	0.521003	0.722527
Model 3: Fully adjusted	0.608962	<0.001	0.508648	0.729059

Model 2 = adjusted for sex, age, body mass index, and smoking status. Model 3 = adjusted for Model 2 + hypertension, and HbA1c. TyG index: Triglyceride to glucose index.

**Table 8:** Association between TyG index and CKD outcomes using restricted cubic spline models.

Outcome	Overall *p*-Value	*p* for Nonlinearity	TyG Percentile (Approx.)	Adjusted OR (95% CI)
Any CKD vs. no CKD	<0.0001	0.44	75th (≈9.75)	1.39 (1.16–1.67)
Early CKD vs. no CKD	0.0026	0.62	75th (≈9.71)	1.27 (1.03–1.57)
Late CKD vs. no CKD	<0.0001	0.30	75th (≈9.72)	1.56 (1.20–2.04)

**Table 9:** ROC analysis results.

Variable Cut-Off Point	AUC (95% CI)	*p*	Sensitivity	Specificity
TyG index > 9.36	0.57 (0.54–0.60)	0.001	0.57	0.55

ROC = receiver operating characteristics; TyG index: Triglyceride to glucose index.

**Table 10:** Regression analysis of the association between TyG index and kidney disease (combined early and late stages) stratified by sex and age group *.

Sex				
Outcome	Male		Female	
	OR (95% CL)	*p*	OR (95% CL)	*p*
Kidney disease	1.328 (0.986–1.788)	0.062	1.831 (1.417–2.365)	<0.001
Age				
Outcome	<60		≥60	
Kidney disease	OR (95% CL)	*p*	OR (95% CL)	*p*
	1.745 (1.341–2.271)	<0.001	1.526 (1.153–2.020)	0.003

* Data presented from the fully adjusted model = adjusted for sex, age, body mass index, smoking status, hypertension, and HbA1c. TyG index: Triglyceride to glucose index.

## Data Availability

Data available upon request.
